# FUNCTIONAL LIMITATION: DIFFERENCES BY EPIGENETIC AGING, ETHNICITY, AND EXPERIENCES OF DISCRIMINATION

**DOI:** 10.1093/geroni/igad104.0183

**Published:** 2023-12-21

**Authors:** Maria Brown, Miriam Mutambudzi

**Affiliations:** Syracuse University, Syracuse, New York, United States; Syracuse University, Syracuse, New York, United States

## Abstract

Epigenetic aging provides an opportunity to rethink processes of aging and policy and health interventions to address health disparities across the life course. Previous research has shown that social factors can result in accelerated epigenetic aging, with subsequent unfavorable health outcomes. This cross-sectional study aimed to evaluate the relationship between epigenetic aging and functional and instrumental limitations in White and Hispanic older adults and to explore how recent experiences of perceived discrimination may differentially impact this association. We used 2014 and 2016 Health and Retirement Study (HRS) data for White (n=1072) and Hispanic (n=139) adults aged 50 and older and epigenetic clock data from the 2016 HRS Venous Blood Study. We hypothesized that accelerated epigenetic aging (PhenoAge epigenetic clock regressed on chronological age) would be associated with greater limitations in activities of daily living (ADLs) and instrumental activities of daily living (iADLs), and this impact would differ by ethnicity and perceived discrimination. On average, Hispanics reported significantly more limitations in ADLs (1.10 vs .47, p<.0001) and iADLs (1.22 vs .72, p<.0001), and more experiences of being treated differently in service situations (44.6% vs 33.3%, p=.0084) and of people acting as if they were afraid of them (28.78% vs 22.01%, p=.0738). Epigenetic age was associated with discrimination (b=-1.98, p=.0078), with ADLs in Hispanics (b=-0.04, p=.0812), and with iADLs in Whites (b=.0069, p=.0443). Further studies of the impact of epigenetic aging on functional and instrumental limitations should be longitudinal, utilize larger samples of older adults, and oversample racial and ethnic minorities.

